# The Synthesis and Biological Activity of Organotin Complexes with Thio-Schiff Bases Bearing Phenol Fragments

**DOI:** 10.3390/ijms24098319

**Published:** 2023-05-05

**Authors:** Ivan V. Smolyaninov, Andrey I. Poddel’sky, Daria A. Burmistrova, Yulia K. Voronina, Nadezhda P. Pomortseva, Maria A. Polovinkina, Nailya R. Almyasheva, Maria A. Zamkova, Nadezhda T. Berberova, Igor L. Eremenko

**Affiliations:** 1 Chemistry Department, Astrakhan State Technical University, 16 Tatisheva Str., 414056 Astrakhan, Russia; 2 Chemistry Department, University of Tübingen, Auf der Morgenstelle 18, 72076 Tübingen, Germany; 3 Kurnakov Institute of General and Inorganic Chemistry, Russian Academy of Sciences, Leninskii Prospekt 31, 119071 Moscow, Russia; 4 Toxicology Research Group of Southern Scientific Centre of Russian Academy of Science, 41 Chekhova Str., 344006 Rostov-on-Don, Russia; 5 Gause Institute of New Antibiotics, 11/1 Bolshaya Pirogovskaya Str., 119021 Moscow, Russia; 6 N.N. Blokhin National Medical Research Center of Oncology, Ministry of Health of the Russian Federation, Kashirskoe sh., 24, 115478 Moscow, Russia

**Keywords:** thio-Schiff bases, organotin complexes, antioxidant activity, radical scavengers, antiproliferative activity

## Abstract

A number of novel di- and triorganotin(IV) complexes **1**–**5** (Ph_2_SnL^1^, Ph_2_SnL^2^, Et_2_SnL^2^, Ph_3_SnL^3^, Ph_3_SnL^4^) with mono- or dianionic forms of thio-Schiff bases containing antioxidant sterically hindered phenol or catechol fragments were synthesized. Compounds **1**–**5** were characterized by ^1^H, ^13^C NMR, IR spectroscopy, and elemental analysis. The molecular structures of complexes **1** and **2** in the crystal state were established by single-crystal X-ray analysis. The antioxidant activity of new complexes as radical scavengers was estimated in DPPH and ABTS assays. It was found that compounds **4** and **5** with free phenol or catechol fragments are more active in these tests than complexes **1**–**3** with tridentate O,N,S-coordinated ligands. The effect of compounds **1**–**5** on the promoted oxidative damage of the DNA by 2,2’-azobis(2-amidinopropane) dihydrochloride and in the process of rat liver (Wistar) homogenate lipid peroxidation in vitro was determined. Complexes **4** and **5** were characterized by more pronounced antioxidant activity in the reaction of lipid peroxidation in vitro than compounds **1**–**3**. The antiproliferative activity of compounds **1**–**5** was investigated against MCF-7, HTC-116, and A-549 cell lines by an MTT test. The values of IC_50_ are significantly affected by the presence of free antioxidant fragments and the coordination site for binding.

## 1. Introduction

One of the simple and useful approaches to multicenter molecules is the synthesis of Schiff bases and the formation of a CH=N linker group bonding different molecular blocks. The presence of an azomethine linker plays an important role in the metal chelation and the bioactivity modulation of metal complexes. The advantage of Schiff bases, as promising ligands in coordination chemistry, is the simplicity of their molecular design, as well as the ability to control steric and electronic effects by the use of various substituents. Metal complexes with Schiff bases combining redox-active phenolic fragments and nitrogen- and sulfur-containing groups or heterocycles possess a wide spectrum of biological activity [1]. In organotin complexes with tridentane O,N,O′, O,N,N′ Schiff bases have recently been addressed since these objects can be used as an easily tuned multichromic system when creating substances with luminescent and fluorescent properties [2,3]. The wide variability of such types of complexes is largely determined by the set of ligands, as well as organic groups at the tin(IV) atom. This approach can be applied not only to regulate photophysical properties but also promote biological activity. Organotin compounds with Schiff bases possessing fluorescence have found an application in the design of materials, solar cells [4,5], and dyes in cancer cell detection [6]. Along with unusual electrochemical and photophysical properties, diorganotin(IV) complexes with O,N,O′ and O,N,N′ Schiff bases have antimicrobial, anti-inflammatory, and antioxidant activities and cytotoxicity against different cancer cell lines [7,8,9,10,11,12,13,14].

Most papers on coordination compounds with tridentate O,N,S donor ligands based on salicylaldehyde are mainly oriented to the synthesis of transition metal complexes and the study of their physical properties [15,16,17,18,19,20]. In addition to the spectral, magnetochemical, and redox properties of such objects, a line of research has recently shifted to the bioinorganic chemistry area due to the wide spectrum of biological activity of related compounds [21,22]. Among the works on diorganotin(IV) derivatives with O,N,S ligands, particular attention focuses on the study of cytotoxicity [23]. However, organotin(IV) compounds with monodentate sulfur donor ligands, such as thiols/thione, have also possessed anticancer, antioxidant, and anti-inflammatory activity [24]. The high toxicity of organotin compounds makes it difficult to develop new metal-based therapeutic agents. It has recently been shown that one of the solutions to this problem is the application of ligands with an antioxidant function, such as sterically hindered phenol, directly bonded to the tin atom or by sulfide linker. This way made it possible to reduce the toxic effect of organotin compounds on healthy cells, as well as reduce side effects [25,26,27,28].

**Scheme 1:**
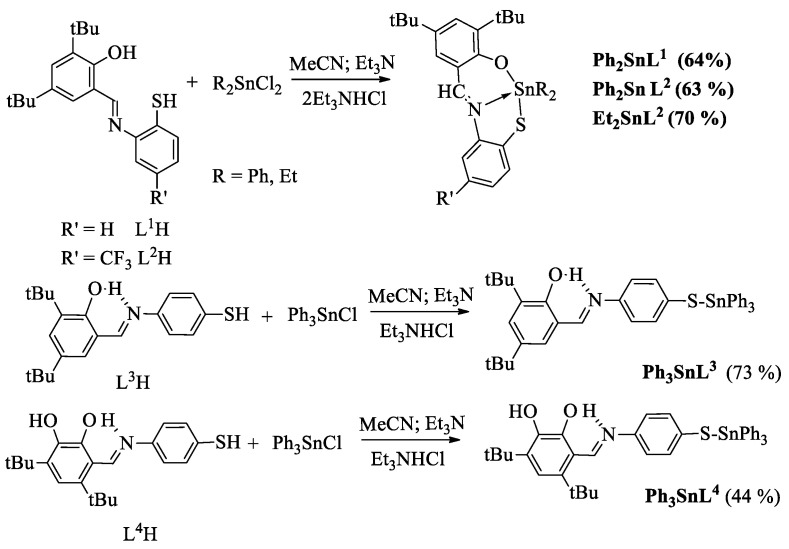
Synthesis of organotin complexes **1**–**5** of the type R_n_SnL (n = 2,3).

In this paper, we provide a comprehensive investigation of di- or triorganotin(IV) complexes with sulfur-containing Schiff bases bearing a free or bonded antioxidant phenol (catechol) group focusing on the bioactivity of these compounds, including anti/prooxidant properties and cytotoxicity in vitro. The main goal of the research is to establish the role of antioxidant fragments and the role of coordination mode on the structure–activity relationship to promote further ligand design with the selection of organic moieties favoring the modulation of organotin(IV) compound cytotoxicity. In the present work, we have carried out the synthesis of new di- and triorganotin(IV) complexes **1**–**5** (Scheme 1) with tri- or monodentate thio-Schiff bases L^n^H with a phenol (catechol) group, and we studied their anti/prooxidant properties and antiproliferative activity.

## 2. Results

### 2.1. Synthesis and Characterization

The target compounds **1**–**5** were obtained by the interaction of Schiff bases L^1^H-L^4^H with organotin chlorides Ph_2_SnCl_2_, Et_2_SnCl_2,_ or Ph_3_SnCl (Scheme 1) in acetonitrile in the presence of 2 or 1 eq. under anaerobic conditions to prevent side oxidation processes of ligands. Compounds **1**–**3** were isolated as yellow-orange crystalline powders with yields up to 70%. Complex **4** is a pale yellow powder, while compound **5** was isolated as a red powder. All compounds are air-stable in the solid state. Compounds have been characterized by IR, ^1^H, and ^13^C{^1^H} NMR spectroscopy, and HR-MS and C,H,N elemental analysis. The spectral and elemental analysis data confirm the composition of complexes. The X-ray-suitable crystals of **1** and **2** were grown by the slow evaporation of acetonitrile in the air. The intense bands of stretching vibrations in the IR spectra of complexes **1**–**5** are observed in the region of 1570–1613 cm^−^^1^, corresponding to stretching vibrations of the C=N bond. The IR spectra of compounds **4** and **5** demonstrate absorption bands in the region of 3430–3501 cm^−1^, appropriating to the stretching vibrations of the O-H bonds of the sterically hindered phenol or catechol groups.

The structures of all complexes and the new ligand L^2^H were confirmed by ^1^H and ^13^C{^1^H} NMR spectroscopy (Figures S1–S12 in Supplementary Materials). The ^1^H NMR spectra of all complexes contain a set of signals corresponding to the functional groups in the complexes’ structures. The signals from imino-group protons in the ^1^H NMR spectra of complexes **1**–**3** (which appear at 8.58 ppm for diphenyltin(IV) derivatives **1** and **2** and at 8.49 ppm for diethyltin(IV) derivative **3**) contain the satellite splitting of the signal on the magnetic ^117/119^Sn nuclei with ^3^J(H,^117/119^Sn) = 48.8, 47.2 and 40.4 Hz, respectively. The splitting points out the coordination of the imino group to the central tin atom. The hydroxyl protons of triphenyltin(IV) p-thiophenolates **4** and **5** give rise to one singlet at 13.65 ppm for **4** and two singlets at 6.38 and 15.80 ppm for **5**. The high values (13.65 and 15.80 ppm) belong to the hydroxyl groups in the 2nd positions of the aromatic phenol or catechol rings involved to the intramolecular hydrogen contacts “-OH…N=CH-” with the neighboring imino-groups.

### 2.2. X-ray Analysis

**Figure 1:**
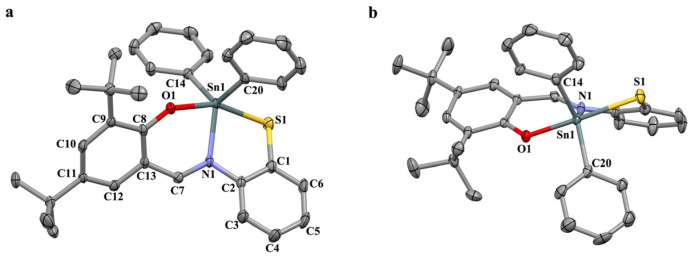
The molecular structure of **1** in the crystal: (**a**) general view; (**b**) view almost along the Sn1-N1 bond. Ellipsoids of 50% probability. The hydrogen atoms are omitted.

**Figure 2:**
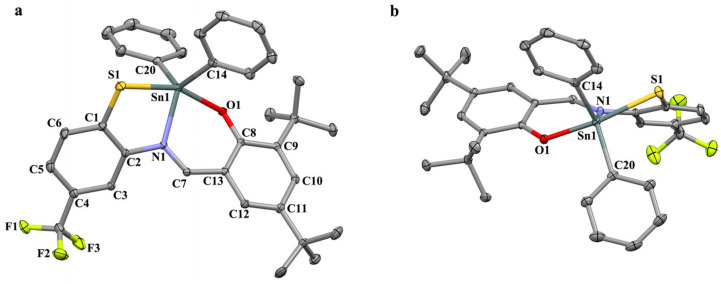
The molecular structure of **2** in the crystal: (**a**) general view; (**b**) view almost along the Sn1-N1 bond. Ellipsoids of 50% probability. The hydrogen atoms are omitted.

Compounds **1** and **2** in crystals are complexes of six coordinated tin (Figure 1 and Figure 2). Polyhedrons are the distorted trigonal bipyramids (τ = 1.509 and 1.607 in **1** and **2**, respectively) formed by atoms S, O, and N in the thio-Schiff bases and two carbon atoms in the phenyl groups. The distribution of bond lengths, valence, and torsion angles in ligands are standard for each type of bond in the related complexes [29,30,31,32,33,34]. The lengths of Sn1-O1, Sn1-N1, and Sn1-S1 bonds are 2.113(2)Å, 2.114(1)Å, 2.163(3)Å, 2.170(1)Å, and 2.536(1)Å 2.547(4)Å in compounds **1** and **2** respectively.

The chelate coordination of Schiff bases occurs in such a way that the chelate cycles are not planar. The bent angle (the bent line is nearly close to the line S1…N1) between planes Sn1S1N1 and S1C1C2N1 is 30.2(2)° in **1** and 32.1(2)° in **2**, and the bent angle (the bent line is nearly close to the line O1…N1) between the planes Sn1O1N1 and O1C8C13C7N1 is 32.2(1)° and 31.6(2)° (Figure 1b and Figure 2b, respectively). The angle formed by six-membered carbon cycles C(1–6) and C(8–13) is equal to 53.3(1)° and 50.4(2)° in **1** and **2,** respectively.

The CCDC contains data on the 84 structures of complexes with similar ligands. Analysis of the geometry of chelate cycles and the structure of coordinated Schiff bases showed that their configuration is generally flatter. The angles between planes Sn1S1N1 and S1C1C2N1 and Sn1O1N1 and O1C8C13C7N1 are less than 20 degrees; the angles between the six-membered carbon cycles are mostly in the range of 0–50 degrees in most parts of the structures. The maximum deviation from the plane is observed in the case of maximally sterically shielded structures. If the deviation from the plane of chelate cycles is due to the coordination of metal ions, then the reversal of six-membered cycles apparently is due to steric factors. A similar situation is observed in complexes of related O,N,O′ chelating ligand data, which is presented in CCDC. Thus, there is a more significant distortion of the tridentate ligand and chelate cycles in the studied complexes compared to the related diorganotin(IV) complexes with similar O,N,O′ presented in the CCD*C* and described by us earlier [35], which is also presented in the CCD*C* O,N,S chelating ligands. The absence of classical centers of hydrogen bonding leads to the fact that the crystal packaging is formed due to weak CH…O, CH…S, CH…π (and CF…π in **2**) interactions forming an infinite three-dimensional structure.

### 2.3. Radical Scavenging Activity

We have explored the radical scavenging activity of complexes **1**–**5** and ligands with the use of different experimental assays, such as the reaction with a diphenylpicrylhydrazyl radical (DPPH) radical, ABTS^●+^ radical cation generated by a potassium persulfate, and the inhibition process of superoxide radical anion formation by xanthine oxidase (NBT assay). A comparative evaluation of radical scavenging properties of compounds **1**–**5** with a known antioxidant, such as Trolox, was performed in a reaction with a 2,2-diphenyl-1-picrylhydrazyl radical. The IC_50_ parameter was determined in dichloromethane at 298 K (Table 1). In the reaction of **1**–**5** with the DPPH radical, it has been found that the value of IC_50_ depended significantly on the presence of free hydroxyl groups in the ligands. For compounds **1**–**3** with the tridentate O,N,S ligand, the IC_50_ parameter significantly exceeds those for the free ligands L^1^H and L^2^H, which indicates a decrease in neutralizing activity. Unlike complexes **1** and **2**, the replacement of acceptor phenyl groups by ethyl ones and the presence of a trifluoromethyl substituent in the ligand contributes to a decrease in the IC_50_ value to 143.7 µM in the case of compound **3**. Similar high IC_50_ indicators were also obtained using ethanol as a medium. Previously studied carboxylate organotin complexes with O,N,N′ chelating Schiff bases are distinguished by greater antiradical activity (IC_50_ ranged from 13 to 114 µM) compared to compounds **1**–**3** [10,36].

Such behavior of complexes **1**–**3** can be explained by the possibility of the formation of relatively stable oxidized forms upon interaction with the DPPH radical and the resulting equilibrium between the monocationic complexes and the reduced form of the radical. At the same time, the interaction of the DPPH radical with the complexes followed by hydrogen atom abstraction is impossible due to the absence of free hydroxyl groups. Such a reaction of electron transfer requires a further stage of proton transfer; however, the required protons are absent in dry CH_2_Cl_2_. As a result, the interaction of complexes with the DPPH radical is complicated.

Complexes **4** and **5** containing free phenol or catechol fragments exhibit a more pronounced antiradical activity in the DPPH test (Table 1). The minimum IC_50_ values were obtained for complex **5**, which confirms its high radical scavenging properties. In contrast to free ligands, the coordination of the thiol group to the tin atom contributes to a decrease in the activity of complexes. The presence of an intramolecular hydrogen bond between the phenolic hydroxyl and the imino group leads to a threefold decrease in the IC_50_ index compared to the complex triphenyltin(IV) 3,5-di-tert-butyl-4-hydroxythiophenolate (IC_50_ = 15.0 ± 4.0) [28].

The interaction of complexes **1**–**3** with the more acceptor ABTS^∙+^ resulted in similar IC_50_ values ranging from 25 to 35 μmol. As in the case of the DPPH radical, the coordination of the phenol group with the R_2_Sn fragment favors an increase in IC_50_ values and, consequently, a radical scavenging activity is reduced. The value of the TEAC parameter decreases by 30–50%. Complexes **4** and **5** are characterized by higher activity: the TEAC index for complex **5** is close to the data obtained for Trolox, and, in the case of compound **4**, it slightly exceeds them.

In this study, we use the NBT assay to evaluate also superoxide radical anion scavenging activity of target compounds. Superoxide radical anion is generated by the xanthine–xanthine oxidase. This intermediate can be reduced by nitro blue tetrazolium (NBT) to the blue-colored (560 nm) formazan. Compounds that inhibit the formazan formation can be considered scavengers of superoxide radical anion. The data for complexes **1**–**3** indicate their high neutralizing activity toward the generated O_2_^∙−^. The scavenging activity decreases in the order from compound **2** to **1** and further to complex **3**. Complexes **1**–**3** have more pronounced antiradical properties compared to free ligands and Trolox (Table 1).

Compounds **4** and **5** demonstrate the opposite effect: a significant increase in the color intensity of solutions is observed in the presence of these complexes. Such behavior points out the superoxide radical anion formation. In blank experiments, the insertion of complexes **4** and **5** into samples in the absence of xanthine oxidase during the incubation of hypoxanthine and formazan did not lead to the coloring of solutions. The complexes act as promoters only when the enzyme is working. Complex **5** containing a catechol group is a more active initiator of superoxide radical anion (254%). Catechol fragment oxidation can cause the o-semiquinone radical anion or o-benzoquinone formation. These species can participate in a redox cycle with the O_2_/O_2_^∙−^ redox couple. Therefore, complexes **4** and **5** can be considered potential inducers of O_2_^∙−^ and other ROS, and one should expect a cytotoxic effect against cancer cell lines.

### 2.4. AAPH-Induced Oxidation of DNA and Lipid Peroxidation

**Figure 3:**
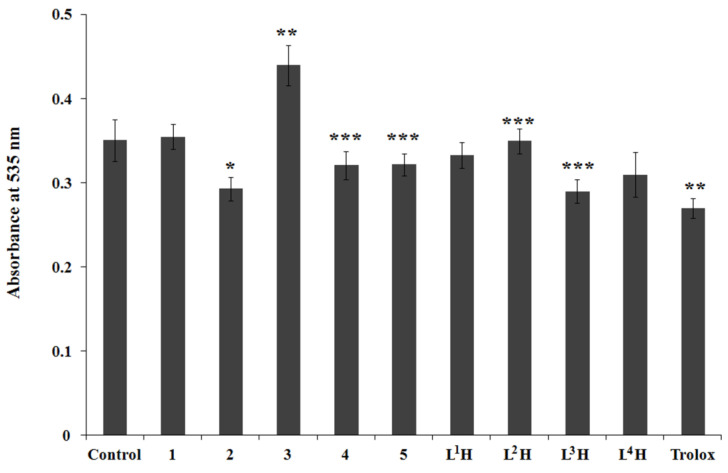
The variety of the absorbance of TBARS in the DNA oxidation (2.0 mg∙mL^−1^) induced by 40 mM AAPH in the presence of complexes **1**–**5**, L^n^H and Trolox (the results are expressed as mean ± standard deviation (* *p* < 0.001; ** *p* < 0.005; *** *p* < 0.01).

The next stage of our research of complexes’ antioxidant activity dealt with the influence of complexes on promoted oxidative damage of the DNA molecules by 2,2′-azobis(2-amidinopropane) dihydrochloride (AAPH). This initiator at a physiological temperature (37 °C) decomposes radical intermediates, such as the peroxide radicals that induce DNA damage. The process leads to the degradation of products that form a colored complex with thiobarbituric acid (TBARS). The target compounds can donate electron or hydrogen atoms, neutralizing peroxyl radicals and preventing the development of DNA oxidative damage. The AAPH-induced oxidation of DNA was carried out with additives of complexes **1**–**5**, ligands L^n^H, and Trolox at a concentration equal to 50 µmol. The absorbance of TBARS in UV-vis spectra was measured after the oxidation for 150 min in comparison with that in the blank experiment (control) (Figure 3).

**Figure 4:**
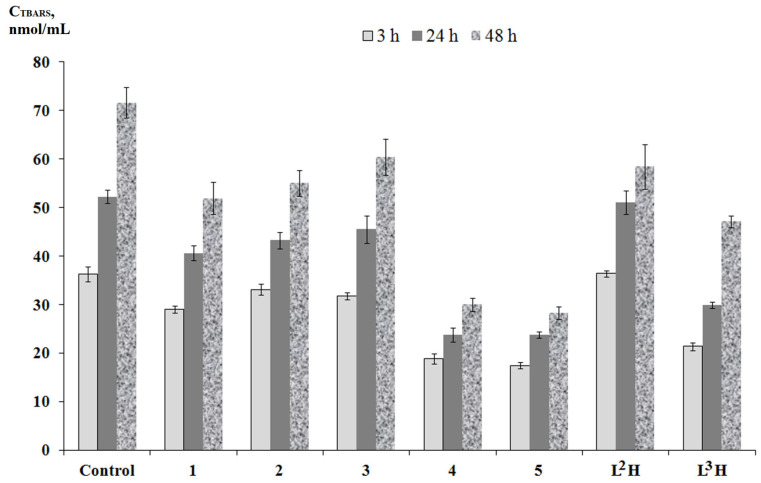
The level of TBARS in rat liver homogenates in vitro in the presence of compounds **1**–**5**, L^2^H, and L^3^H, and without additives (control) during the incubation at 3, 24, and 48 h (the concentration of compounds in the reaction medium is 0.1 mM). The results are expressed as mean ± standard deviation (*p* < 0.001).

In the presence of compounds **2**, **4**, and **5** or L^1^H, L^3^H, and L^4^H, the absorbances of TBARS are lower than those in the blank experiment (Figure 4, control). This fact points out the inhibiting effect of these substances on DNA degradation. Complexes **2**, **4**, and **5** act as weak antioxidants, reducing the TBARS content by 8–17% in this test. Schiff bases L^3^H and L^4^H possess more pronounced inhibition than L^1^H, while the L^2^H ligand does not demonstrate any effect on the level of TBARS. The same behavior is proper to complex **1**. Complex **2** with ethyl group at the tin atom is characterized by the minor promotion action on the process of DNA oxidation. The promotion effect can be explained by the possibility of the labile Sn-C bond breaking upon interaction with peroxyl radicals and the subsequent formation of an active alkyl radical. Reactions of this type are characteristic of organometallic derivatives of tin(IV) upon the interaction with peroxyl radicals [37], and ones were also observed under electrochemical activation of tin complexes with redox-active ligands [38].

We have previously shown that lipophilic organotin compounds can act as prooxidants, promoting lipid peroxidation processes in vitro that can exacerbate oxidative stress [39]. The reaction of organotin compounds with peroxyl radicals and molecular oxygen promotes the formation of active radicals due to the homolytic cleavage of Sn-C bonds [40]. One of the ways to prevent the toxic effect of organotin compounds is either the addition of antioxidants together with R_3_SnX and R_2_SnX_2_ or the chelation of the organometallic fragment due to the formation of covalent or coordinated bonds with various types of ligands possessing antiradical and antioxidant activity [25,41].

At this research stage, it is interesting to evaluate the influence of target organotin complexes on the lipid peroxidation reaction of the rat (Wistar) liver homogenate as a non-enzymatic process induced by Fe(II) ions (in vitro). The lipid peroxidation of the rat liver homogenates was assessed by the accumulation of TBARS products. The samples of the rat brain homogenates were divided as follows: one control (blank experiment) and homogenates with additives of complexes **1**–**5** and ligands L^2^H and L^3^H. TBARS concentrations were determined by measuring the absorbance of the solution at 535 nm using UV-vis spectroscopy (Figure 4).

All complexes possess an antioxidant activity reducing the concentration of TBARS compared to the control experiment. These data are consistent with the previously obtained results for related organotin compounds with Schiff base ligands [9,36]. The inhibition effect for these compounds is considerably different for complexes **1**–**3, 4** and **5**. In general, compounds **1**–**3** were less active LP inhibitors, lowering the TBARS content by 8–27%. The effectiveness of the antioxidant action increased slightly during the incubation time. However, complexes **2** and **3** have a more pronounced antioxidant effect on lipid peroxidation than the free L^2^H ligand. In contrast, compounds **4** and **5** with free phenol or catechol fragments have been characterized with more marked antioxidant properties, reducing the level of TBARS from 48 to 60%. In contrast to complex **4**, the effectiveness of the antioxidant action of Schiff base L^3^H decreased with the elongation of the incubation period from 41 to 34%. In the case of these complexes, the TBARS content decreases gradually during the incubation time. Consequently, the presence of free phenolic and catecholic groups acting as hydrogen atom donors causes the neutralizing activity of the complexes toward ROO radicals and lipid hydroperoxides. Complexes exhibit a prolonged antioxidant action when the concentration of lipid peroxidation products increases significantly with time for the control experiment, and more effective stabilization of the LP process was observed in the case of compounds **4** and **5**.

### 2.5. Antiproliferative Activity

The antiproliferative activity of compounds **1**–**5** was also studied. The compounds were investigated against A-549 (adenocarcinoma human alveolar basal epithelial), HCT-116 (human colon cancer), and MCF-7 (human breast cancer) cells using the MTT test. The IC_50_ values were determined in comparison with Doxorubicin (Table 2).

A significant difference in the IC_50_ values is observed for complexes **1**–**3**, **4**, and **5** (Table 2). The concentration data for complexes differ by two orders of magnitude. A number of compounds, **1**–**3,** also have differences: the presence of a trifluoromethyl group in the aromatic ring of the ligand contributes to a decrease in the IC_50_ values for **2** compared to **1**. The substitution of phenyl by ethyl groups in the tin atom has the same effect in the case of **3**. Five-coordinate tin complexes with O,N,S donor Schiff bases are less cytotoxic than related complexes containing O,N,O′ ligands [6,11,14]. The exchange of one oxygen atom by sulfur in the coordination site contributes to a significant increase in the IC_50_ values, as well as a decrease in the toxic effect of the organometallic fragment. Ligands of this type can be considered potential chelators of organotin compounds, promoting the reduction of their toxic effect. The cytotoxicity parameters for **4** and **5** are close to doxorubicine IC_50_. These values significantly exceed cisplatin data (Table 2). The IC_50_ indexes for **4** and **5** are observed in a narrow range of values. In the presence of complexes **4** and **5**, a greater sensitivity was determined against HTC-116 cancer cells.

Compared to the previously studied free ligands L^3^H and L^4^H, the binding of the Schiff base thiol group to the triphenyltin(IV) fragment reduces the IC_50_ values by two orders of magnitude, which suggests a significant increase in the cytotoxic effect of the complexes. However, the IC_50_ values for complexes **4** and **5** toward the HCT-116 and MCF 7 cell lines are 2–5 times higher than the data (0.06–0.09 µM) for the triphenyltin complex bound to the phenolic antioxidant group [28]. The cytotoxicity indices are significantly affected by the coordination accessibility of the tin atom. In the case of compounds **4** and **5**, the presence of an additional coordination site significantly enhances the ability of five-coordination compounds **1**–**3** to bind to various biomolecules.

## 3. Materials and Methods

### 3.1. General

The starting reagents were commercially available, including 3,5-di-tert-butylsalicylic aldehyde («Acros Organics», 99%, Shanghai, China), 2-amino-4-(trifluoromethyl)benzenethiol hydrochloride («Alfa Aesar», 97%, Heysham, UK), 2-aminothiophenol (≥97%, Hohenbrunn, Germany), Ph_2_SnCl_2_ («Aldrich», 96%, Munich, Germany), Et_2_SnCl_2_ («Aldrich», 98%), Ph_3_SnCl («Aldrich», 97%), 2,2′-azobis(2-amidinopropane) dihydrochloride (AAPH) (97%, Aldrich, Munich, Germany), 2,2′-azino-bis(3-ethylbenzothiazoline-6-sulfonic acid) (≥98%, TCI, Tokyo, Japan), 2,2-diphenyl-1-picrylhydrazyl (98%, Aldrich), thiobarbituric acid (≥98%, Merck, Darmstadt, Germany), deoxyribonucleic acid sodium salt (DNA) from salmon testes (Sigma-Aldrich, St. Louis, MO, USA), phosphate-buffered saline (PBS) pH 7.4 (Sigma-Aldrich, St. Louis, MO, USA), xanthine oxidase from bovine (≥99%, Sigma-Aldrich, St. Louis, MO, USA), nitroblue tetrazolium (90%, Alfa Aesar, Kandel, Germany), xanthine (≥99%, Sigma-Aldrich, St. Louis, MO, USA), bovine serum albumin (≥96%, Sigma-Aldrich, St. Louis, MO, USA), 3,5-di-tert-butylcatechol (98%, Aldrich), 6-hydroxy-2,5,7,8-tetramethylchromane-2-carboxylic acid (Trolox) (97%, Aldrich), trichloroacetic acid (≥99%, Sigma-Aldrich, Taufkirchen, Germany), EDTA, nitroblue tetrazolium, and xanthine oxidase (0.04 MU) (Aldrich), and were used without further purification in the synthesis of the target compounds and biological tests. Solvents were purified following standard procedures [44].

The IR spectra were recorded on an FSM-1201 FT-IR spectrometer (LLC “Monitoring”, Saint Petersburg, Russia) in KBr pellets. The NMR spectra were measured in CDCl_3_ on Bruker Avance HD 400 spectrometers (Bruker Biospin AG, Faellanden, Switzerland) with a frequency of 400 MHz (^1^H) and 100 MHz (^13^C) using Me_4_Si as an internal standard. The chemical shift values are given in ppm with reference to the solvent, and the coupling constants (J) are given in Hz. The elemental analysis was carried out on a Euro EA 3000 (C,H,N) elemental analyzer (EuroVector Srl, Redavalle, Italy). Mass spectra (HRMS) were recorded on a Bruker UHR-TOF Maxis™ Impact mass spectrometer (Bruker Daltonik GmbH, Bremen, Germany). The UV-VIS spectra were recorded with an SF-104 spectrophotometer (AKVILON, Podol’sk, Russia) in a range of 300–600 nm or a Multiskan Sky microplate spectrophotometer (Thermo Scientific, Waltham, MA, USA).

The X-ray diffraction data for crystals **1** and **2** were collected at 150 K on a Bruker D8 Venture diffractometer equipped with a CCD detector (Mo-Kα, λ = 0.71073 Å, graphite monochromator). Semi-empirical absorption correction was applied by the SADABS program [45]. The structures were solved by direct methods and refined by the full-matrix least squares in the anisotropic approximation for non-hydrogen atoms. The calculations were carried out by the SHELX-2014 program package [46] using Olex2 1.2 [47]. Crystallographic data for structures reported in this paper have been deposited in the Cambridge Crystallographic Data Center (2253131, 2253132).

Crystal data for **1**: C_33_H_35_NOSSn, Mr = 612.37, yellow crystal, monoclinic, space group P2_1_/c, Z = 4, a = 10.1933 (3), b = 18.6781 (6), c = 15.2915 (5) Å, β = 90.297(1)˚, V = 2911.33 (16)Å^3^, ρ_calc_ = 1.397 g/cm^3^, μ = 0.975 mm^−^^1^, 11,935 reflections collected (h, k, l), 5646 independent (Rint 0.0919) and 4289 observed reflections [I > 2σ(I)], 341 refined parameters, R1 = 0.0458, wR2 = 0.1201, the max. residual electron density is 2.21, and (−1.681) eÅ^−^^3^.

Crystal data for **2**: C_34_H_34_F_3_NOSSn, M = 680.37, orange crystal, monoclinic, space group P2_1_/n, Z = 4, a = 9.6295 (3), b = 29.393 (1), c = 11.1420 (4) Å, β = 102.582 (1)˚, V = 3077.91 (18)Å^3^, ρ_calc_ = 1.468 g/cm^3^, μ = 0.94 mm^−^^1^, 31,462 reflections collected (h, k, l), 8166 independent (Rint 0.025) and 7336 observed reflections [I > 2σ(I)], 376 refined parameters, R1= 0.0230, wR2 = 0.0514, the max. residual electron density is 0.34, and (−0.54) eÅ^−^^3^.

### 3.2. Syntheses

The Schiff bases such as 2-(2-mercaptophenylimino)methyl)-4,6-di-tert-butylphenol (L^1^SH), 2,4-di-tert-butyl-6-((4-mercaptophenylimino)methyl)phenol (L^3^H), and 4,6-di-tert-butyl-3-((4-mercaptophenylimino)methyl)catechol (L^4^H) were synthesized by the known methods [15,42]. The reaction of 3,5-di-tert-butyl-2-hydroxybenzaldehyde (4 mmol) with 2-amino-4-(trifluoromethyl)benzenethiol hydrochloride (4 mmol) was carried out in methanol (20 mL). The appropriate hydrochloride with 1 eq. triethylamine (in 1 mL methanol) was added dropwise to a solution of aldehyde (in 20 mL) for 30 min, and then the solution was refluxed for 2 h under argon. The solution was slowly cooled to room temperature. The resulting white precipitate was filtered off and dried under reduced pressure. The yield was 1.02 g, with 2.5 mmol (63%). During the reaction of the aldehyde with *o*-aminothiophenol, a cyclic benzothiazoline form is obtained, and then, in the presence of a base, it transforms into the iminothiol form.

(L^2^H) 2,4-di-tert-butyl-6-(5-(trifluoromethyl)-2,3-dihydrobenzo[d]thiazol-2-yl)phenol IR (KBr, ν/cm^−^^1^): 3293, 3135, 3294, 2909, 2871, 1604, 1580, 1481, 1440, 1395, 1365, 1327, 1311, 1259, 1228, 1161, 1138, 1118, and 1073. ^1^H NMR (CDCl_3_, J, 400 Hz; δ, ppm): 1.29 (s, 9 H, tBu), 1.44 (s, 9H, tBu), 4.72 (br.s, 2H, NH), 6.61 (s, 1 H, CH), 6.91 (m, 1 H, arom. C_6_H_2_), 6.95 (br.s., 1 H, arom. C_6_H_3_), 7.11 (br.d., ^3^J(H,H) = 8.0, 1 H, arom. C_6_H_3_), 7.17 (br.d., ^3^J(H,H) = 8.0, 1 H, arom. C_6_H_3_), 7.37 (m, 1 H, arom. C_6_H_2_), and 8.68 (br.s., 1 H, OH). ^13^C{^1^H} NMR (CDCl_3_, 100 MHz, δ, ppm): 29.66, 31.52, 34.20, 35.20, 73.71, 107.53 (q, ^3^J(C,F) = 3.7), 117.99, 119.23 (q, ^3^J(C,F) = 3.9), 122.16, 123.86, 124.03 (q, ^1^J(C,F) = 271.8), 125.72, 128.01 (q, ^2^J(C,F) = 32.5),133.56, 137.81, 141.47, 145.63, and 153.93. HR-MS: Found *m*/*z*: 408.1623 [M−H]^+^. C_22_H_25_F_3_NOS. Calcd *m*/*z*: 408.1614.

The synthesis of organotin complexes R_2_SnL^n^ (**1**–**3**) was carried out as follows: 2 equiv. of R_2_SnCl_2_ (0.2 mmol) dissolved in acetonitrile (3 mL) was added to a solution of ligands (0.2 mmol) in the same solvent (5 mL), under argon. Further, 2 equiv. of triethylamine was added to the solution under extensive stirring. During the reaction, the initial solution color changed to intense orange. The resulting mixture was left for 2 days at 0–5 °C. The resulting colored precipitates of the complexes were filtered off and dried under reduced pressure. Crystals suitable for X-ray diffraction analysis were obtained by recrystallization of the compounds in acetonitrile. In the case of compounds Ph_3_SL^n^ (**4** and **5**), the reaction was carried out in a similar way in acetonitrile in the presence of 1 equiv. of triethylamine. The resulting reaction mixture was evaporated to 2 mL and cooled at 5 °C for 24 h. The resulting powdery precipitates of complexes **4** and **5** were filtered off, washed with cold acetonitrile, and dried in a vacuum.

Complex Ph_2_SnL^1^ (**1**). The yield of **1** in the form of yellow-orange crystals was 64% (0.077 g). IR (KBr, ν/cm^−^^1^): 3063, 2955, 2905, 2868, 1606, 1580, 1526, 1460, 1459, 1423, 1378, 1318, 1241,1166,1127, and 1065. ^1^H NMR (CDCl_3_, 400 MHz, δ/J, ppm/Hz): 1.32 and 1.44 (both s., both 9 H, tBu), 7.01–7.08 (m., 1 H, arom. C_6_H_4_), 7.11 (d., ^4^J(H,H) = 2.6 Hz, 1 H, arom. C_6_H_2_), 7.12–7.19 (m., 2 H, arom. C_6_H_4_), 7.28–7.42 (m., 6 H, Ph), 7.56–7.61 (m., 1 H, arom. C_6_H_4_), 7.62 (d., ^4^J(H,H) = 2.6 Hz, 1 H, arom. C_6_H_2_), 7.82–7.90 (m., 4 H, satellites, ^3^J(H,^117/119^Sn) = 79 Hz, Ph), and 8.58 (s., 1 H, satellites, ^3^J(H,Sn) = 48.8 Hz, CH=N). ^13^C NMR (CDCl_3_, 100 MHz, δ, ppm): 29.80, 31.15, 34.07, 35.37, 117.54, 118.90 (satellites, J(C,Sn) = 15.2 Hz), 124.52, 127.88, 128.40 (satellites, J(C,Sn) = 81.5 Hz), 129.26, 129.64 (satellites, J(C,Sn) = 16.6 Hz), 131.68, 132.99, 135.91 (satellites, J(C,Sn) = 58.3 Hz), 136.88, 139.16, 141.20, 142.34, 142.91, 167.42, and 169.08. Calcd. For C_33_H_35_NOSSn (%): C, 64.72; H, 5.76; and N, 2.29. Found (%): C, 64.85; H, 5.90; and N, 2.25.

Complex Ph_2_SnL^2^ (**2**). The yield of **2** in the form of orange crystals was 63% (0.086 g). IR (KBr, ν/cm^−^^1^): 3050, 2958, 2908, 2871, 1583, 1528, 1460, 1420, 1358, 1321, 1253, 1170, 1124, 1081. ^1^H NMR (CDCl_3_, 400 MHz, δ/J, ppm/Hz): 1.33 and 1.43 (both s., both 9 H, tBu), 7.15 (d., ^4^J(H,H) = 2.6 Hz, 1 H, arom. C_6_H_2_), 7.28–7.44 (m., 8H: 6 H, Ph + 2 H, arom. C_6_H_3_), 7.65 (d., ^4^J(H,H) = 2.6 Hz, 1 H, arom. C_6_H_2_), 7.66–7.70 (m., 1 H, arom. C_6_H_3_), 7.80–7.87 (m., 4 H, satellites, ^3^J(H,^117/119^Sn) = 79 Hz, Ph), 8.58 (s., 1 H, satellites, ^3^J(H,Sn) = 47.2 Hz, CH=N). ^13^C NMR (CDCl_3_, 100 MHz, δ, ppm): 29.79, 31.10, 34.14, 35.39, 115.66 (m., due CF_3_), 117.54, 123.90 (m., due CF_3_), 128.56 (satellites, J(C,Sn) = 84.0 Hz), 129.47, 129.92, 132.00, 133.93, 135.87 (satellites, J(C,Sn) = 59.0 Hz), 136.27, 139.67, 141.38, 141.64, 143.15, 168.12, 169.64 (m., due CF_3_). Calcd. For C_34_H_34_F_3_NOSSn (%): C, 60.02; H, 5.04; N, 2.06. Found (%): C, 60.13; H, 5.27; N, 2.01.

Complex Et_2_SnL^2^ (**3**). The yield of **3** in the form of yellow crystalline powder was 70% (0.082 g). IR (KBr, ν/cm^−^^1^): 2957, 2908, 2871, 1577, 1528, 1460, 1417, 1383, 1318, 1256, 1173, 1115, and 1084. ^1^H NMR (CDCl_3_, 400 MHz, δ/J, ppm/Hz): 1.28 (t.m., ^3^J(H,H) = 7.8 Hz, satellites 3J(H,^117/119^Sn) = 66.9/64.0 Hz, 6 H, CH_3_ of Et), 1.32 and 1.42 (both s., both 9 H, tBu), 1.44–1.54 (q.m., 4 H, CH_2_ of Et), 7.11 (d., ^4^J(H,H) = 2.6 Hz, 1 H, arom. C_6_H_2_), 7.32–7.38 (m., 2 H, arom. C_6_H_3_), 7.58 (d., ^4^J(H,H) = 2.6 Hz, 1 H, arom. C_6_H_2_), 7.58–7.63 (d.m., ^3^J(H,H) = 8.0 Hz, 1 H, arom. C_6_H_3_), and 8.49 (s., 1 H, satellites, ^3^J(H,Sn) = 40.4 Hz, CH=N). ^13^C NMR (CDCl_3_, 100 MHz, δ, ppm): 9.99, 16.45, 29.43, 31.13, 34.10, 35.28, 115.59 (m., due CF_3_), 117.44, 123.69 (m., due CF_3_), 126.13, 129.00, 132.30, 133.22, 138.96, 141.48, 144.09, 144.23, 167.87, and 169.70. Calcd. for C_26_H_34_F_3_NOSSn (%): C, 53.44; H, 5.87; and N, 2.40. Found (%): C, 53.51; H, 6.02; and N, 2.35.

Complex Ph_3_SnL^3^ (**4**). The yield of **4** in the form of pale yellow powder was 70% (0.100 g). IR (KBr, ν/cm^−^^1^): 3073, 3050, 2960, 2909, 2867, 1613, 1596, 1574, 1482, 1464, 1438, 1430, 1392, 1360, 1332, 1270, 1249, 1197, 1171, 1103, 1074, and 1021. ^1^H NMR (CDCl_3_, 400 MHz, δ/J, ppm/Hz): 1.33 and 1.47 (both s, both 9H, 2 tBu), 6.95 (d, ^3^J(H,H) = 8.5 Hz, 2 H, arom. C_6_H_4_), 7.18 (d, ^4^J(H,H) = 2.3 Hz, 1 H, arom. C_6_H_2_), 7.31 (d, ^3^J(H,H) = 8.5 Hz, 2 H, arom. C_6_H_4_), 7.37–7.52 (m, 9 H, Ph), 7.45 (d, ^4^J(H,H) = 2.3 Hz, 1H, arom. C_6_H_2_), 7.52–7.65 (m, 6 H, Ph), 8.53 (s, 1H, CH=N), and 13.65 (s, 1 H, OH). ^13^C NMR (CDCl_3_, 100 MHz, δ, ppm): 29.38, 31.44, 34.15, 35.06, 118.21, 121.27, 126.71, 128.00, 128.87 (J(C,^117,119^Sn) = 58.4 Hz), 129.14, 129.84 (J(C, ^117,119^Sn) = 12.5 Hz), 135.85 (J(C,^117,119^Sn) = 13.6 Hz), 136.12, 136.68 (J(C,^117,119^Sn) = 42.9 Hz), 137.36, 140.55, 146.80, 158.19, and 163.13. HR-MS. Found *m*/*z*: 692.2020 [M + H]^+^. C_39_H_42_NOSSn. Calcd. *m*/*z*: 692.2010.

Complex Ph_3_SnL^4^ (**5**). The yield of **5** in the form of pale yellow powder was 44% (0.100 g). IR (KBr, ν/cm^−^^1^): 3501, 3467, 3064, 3036, 2954, 2906, 2870, 1602, 1560, 1479, 1429, 1417, 1386, 1365, 1296, 1266, 1221, 1166, 1073, and 1021. NMR (CDCl_3_, 400 MHz, δ/J, ppm/Hz): 1.44 and 1.49 (both s., both 9 H, tBu), 6.38 (s, 1H, OH), 6.80 (s, 1 H, arom. C_6_H_1_), 6.92 (d, ^3^J(H,H) = 8.3 Hz, 2 H, arom. C_6_H_4_), 7.34 (d, ^3^J(H,H) = 8.3 Hz, 2 H, arom. C_6_H_4_), 7.37–7.46 (m, 9 H, Ph), 7.48–7.66 (m, 6 H, Ph), 9.24 (s, 1 H, CH=N), and 15.80 (s, 1 H, OH). ^13^C NMR (CDCl_3_, 100 MHz, δ, ppm): 29.14, 33.30, 35.26, 35.61, 112.67, 113.88, 120.82, 128.88 (J(C,^117,119^Sn) = 58.5 Hz), 129.87 (J(C,^117,119^Sn) = 12.8 Hz), 131.16, 136.12 (J(C,^117,119^Sn) = 13.6 Hz), 136.66 (J(C,^117,119^Sn) = 42.7 Hz), 137.15, 137.27, 140.39, 142.36, 144.96, 154.75, and 160.78. HR-MS. Found *m*/*z*: 706.1786 [M-H]^+^. C_39_H_40_NO_2_SSn. Calcd. *m*/*z*: 706.1814.

### 3.3. The DPPH Radical Scavenging Activity Assay

DPPH radical scavenging activity was performed according to the method of Bondet et al. [48] with some modifications [49]. A solution of the radical DPPH in CH_2_Cl_2_ (C_0_ = 50 µM) was prepared daily and protected from light. An absorbance was recorded to check the stability of the radical throughout the time of analysis. The solution of the tin complex in CH_2_Cl_2_ (0.02 mL) was added to 2 mL of a 50 µM solution of DPPH in CH_2_Cl_2_. The decrease in absorbance was determined at 527 nm (ε_max_ = 1.67∙10^5^ M^−^^1^∙cm^−^^1^) every 5 min until the reaction reached a plateau at room temperature after 1 h. The parameter IC_50_ is the concentration of an antioxidant necessary for decreasing the amount of the DPPH radical by 50% of the initial value. To determine IC_50_, the plot of the residual concentration of the stable radical versus the molar ratio (expressed as the number of moles of the complex per 1 mole of the stable radical) was constructed. All experiments were performed in triplicate at room temperature.

### 3.4. ABTS Assay

The radical cation ABTS^∙+^ is generated by the oxidation of 2,2′-azino-bis(3-ethylbenzothiazoline-6-sulfonic acid) with the K_2_S_2_O_8_. Lowering the intensity of the greenish coloration characteristic of this radical cation reflects the ability of the antioxidants to scavenge this species [50]. ABTS was dissolved in water to a 7 mM concentration. The generation of radical cation was produced by reacting the ABTS stock solution with 2.45 mM K_2_S_2_O_8_ (final concentration) and allowing the mixture to stand in the dark at room temperature for 12–20 h before use. A solution of ABTS^•+^ has an absorbance with a maximum of 734 nm. Stock solutions of test compounds and Trolox with a concentration of 1.0 mM were prepared in DMSO. A 7 mM ABTS solution (40 μL) was added to 2.5 mL of ethanol, and the absorbance was measured at 734 nm at room temperature. The value of absorbance was in the range of 0.70–0.72 (A_0_). Then, various aliquots of substances were added, and the final concentration varied from 1 to 40 µM. The absorbance (A_i_) reading was taken at room temperature after initial mixing once a minute for 6 min. All measurements were carried out at least three times. Plots of absorbance versus concentration were prepared for test compounds and Trolox. TEAC values were measured by comparing the slopes of plots obtained for each complex compared to that of Trolox. The absorbance of the blank (40 μL DMSO and 40 μL of radical cation) assay was set as 100% radical. The IC_50_ values were calculated as the minimum concentration of each sample required to inhibit 50% of the ABTS radical cation.

### 3.5. Inhibition of Superoxide Radical Anion Formation by Xanthine Oxidase (NBT Assay)

To evaluate the radical scavenging activity of tin complexes and ligands, a reaction with O_2_^∙−^ generated in the xanthine/xanthiooxidase enzymatic system was used. This reaction was based on the ability of tetrazolium blue to be reduced to formazan upon interaction with the superoxide anion radical. The reaction mixture consisted of 2.70 mL of a 40 mM sodium carbonate buffer containing 0.1 mM EDTA (pH 10.0), 0.06 mL of 10 mM xanthine, 0.03 mL of 0.5% *w/v* bovine serum albumin, 0.03 mL of 2.5 mM nitroblue tetrazolium, and 0.06 mL of the sample solution in DMSO. A total of 0.05 mL of xanthine oxidase (0.04 units) was added to the mixture at 22 °C, and the absorbance at 560 nm (by the formation of blue formazan) [51] was recorded by a Thermo Scientific Multiskan Sky microplate spectrophotometer for 800 s. A control experiment was carried out by replacing the sample solution with the same amount of DMSO. Inhibition I (%) = [(1 − A_i_/A_0_) × 100%], where A_i_ is the absorbance in the presence of target compounds at the end of the reaction (800 s) and A_0_ is the absorbance of the blank solution. The IC_50_ values were determined graphically using the dependence of percent inhibition values versus the concentration of the compound. All experiments were performed three times.

### 3.6. AAPH-Induced Oxidation of the DNA Assay

AAPH-induced oxidation of DNA (the deoxyribonucleic acid sodium salt from salmon testes) was carried out following the known method with a little modification [52]. Briefly, 0.02 mL of stock solutions of the research compounds in DMSO were added to PBS (pH 7.4) solutions of 2,2′-azobis(2-amidinopropane) dihydrochloride and DNA. The final concentration of DNA and AAPH was kept at 2.5 mg∙mL^−^^1^ and 40 mmol, respectively. Then, the above solution was dispatched into test tubes with a 2.0 mL solution each. All the tubes were incubated in a water bath for 2.5 h at 37 °C to initiate oxidation. Test tubes were taken out and cooled immediately, to which 1.0 mL of TBA (1.00 g TBA and 0.40 g NaOH dissolved in 100 mL PBS (pH 7.4)) and 1.0 mL of 3.0% trichloroacetic acid aqueous solution was added. The tubes were heated in a boiling water bath for 15 min. After cooling, 2.0 mL of n-butanol was added and shaken vigorously to extract TBARS. The absorbance of the n-butanol layer was measured at 535 nm. Finally, the average value of three absorbance data (within 10% experimental error) was determined. The absorbance in the blank experiment was assigned as A_0_, while the absorbances in the presence of the compounds **1**–**5**, ligands L^n^H, and Trolox were assigned as A_i_. The antioxidant effect of the tested compounds (in the percentage of forming TBARS) on the AAPH-induced oxidation of DNA was expressed by (1 − A_i_/A_0_) × 100.

### 3.7. Lipid Peroxidation of Rat Liver Homogenate

Samples of Wistar rat liver were homogenized (1:10 *w*/*v*) in a phosphate buffer, pH 7.4, using a homogenizer. The level of lipid peroxidation was estimated by using the thiobarbituric acid reactive substances (TBARS) assay [53]. The influence of compounds on lipid peroxidation of the rat liver homogenates was carried out at 37 °C in a phosphate buffer (pH 7.4) in the presence or absence of test compounds or vehicles (CHCl_3_). The concentration of compounds in the medium was 0.1 mM. The level of lipid peroxidation was measured as a non-enzymatic process by the addition of ascorbic acid and (NH_4_)_2_Fe(SO_4_)_2_. The homogenate was divided into the following experimental groups: one control homogenate and seven samples of homogenate with the addition of target compounds. Solutions of ascorbic acid (0.1 mL, 2.6 mmol), Fe(NH_4_)_2_(SO_4_)_2_ (0.1 mL, 0.4 mM), and trichloroacetic acid (1 mL, 40%) were injected into the probe. The test tubes were incubated at 37 °C and then probes were centrifuged (10 min at 4000× *g*). The supernatants (2 mL) were transferred to new test tubes and mixed with 1 mL of TBA solution (0.8%). The probes were heated at 95 °C for 10 min and cooled at 4 °C, and then the probes were centrifuged (10 min at 10,000× *g*) and the absorbance of the supernatants was measured at 535 nm using a Thermo Scientific Multiskan Sky microplate spectrophotometer. TBARS concentrations were determined after 3, 24, and 48 h of incubation. The homogenates were incubated at 0–4 °C between measurements. All the experiments were performed using three independent experiments. Preliminary experiments were performed in the absence of compound interaction with thiobarbituric acid. The values were expressed as mean % ± SD.

### 3.8. In Vitro Cytotoxicity Assay

Human breast cancer cell line (MCF-7), lung cancer cell line (A549), and colorectal cancer cell line (HCT-116) were obtained from American Type Culture Collection (ATCC, Manassas, VA, USA). Cells were cultivated in Dulbecco’s Modified Eagle’s Medium (Paneko, Moscow, Russia) supplemented with 10% fetal bovine serum (Hyclone, Cytiva Europe GmbH, Wien, Austria), 2 mM L-glutamine (Paneko, Moscow, Russia), 1% penicillin (Paneko, Moscow, Russia), and 1 % streptomycin (Paneko, Russia) at 37 °C and 5% CO_2_. Tin complexes **1**–**5** were dissolved in DMSO at a starting concentration of 10 mM, followed by serial dilutions in a cultural medium before the experiments. The final DMSO concentration was lower than 0.1% and did not affect the viability of the cells.

Cell viability after exposure to compounds **1**–**5** was determined by the MTT (3-(4,5-dimethylthiazol-2-yl)-2,5-diphenyl tetrazolium bromide) assay. The cells (5 × 10^3^ in 190 μL of culture medium) were seeded into 96-well plates for 24 h and treated with compounds (**1**–**5**) in concentrations ranging from 0.10 to 400.00 μM for 72 h. After treatment with test compounds, 10 μL (5.00 mg/mL) of the MTT reagent (Paneko, Moscow, Russia) was added into each well for 1 h. After incubation, the medium was discarded, 200 μL of DMSO was added, and the absorbance at 540 nm was measured. The value of IC_50_, which represents the concentration of compound required to reduce the viability of cells at 50% compared to control cell growth (100%), was determined. Each assay was performed in triplicate in at least two separate experiments. Additionally, DMSO at a concentration of 0.1% was used as a negative control and doxorubicin hydrochloride (Sigma-Aldrich, St. Louis, MO, USA) was used as a positive control.

## 4. Conclusions

The interaction of thio-Schiff bases bearing phenol fragments with di- or triorganotin chlorides leads to the formation of target complexes with a preparative yield of up to 73%. The molecular structures of compounds **1** and **2** in crystals were studied by X-ray diffraction. Compounds **1** and **2** are complexes of six-coordinated tin. Polyhedrons are the distorted trigonal bipyramids formed by S, O, and N atoms of the thio-Schiff bases and two carbon atoms of phenyl rings.

Complexes **4** and **5** with free phenol or catechol groups demonstrate more a pronounced radical scavenging activity in DPPH and ABTS assays. At the same time, these compounds have a promotion effect on O_2_^∙−^ generation. In contrast, organotin complexes **1**–**3** with O,N,S ligands play the role of superoxide radical anion scavengers. The antioxidant activity of most complexes in the process of DNA oxidative damage was weak, and complex **3** acted as a prooxidant. In the presence of target compounds, a non-enzymatic lipid peroxidation of rat liver (Wistar, Philadelphia, PA, USA) homogenate is inhibited. Compounds **4** and **5** with free phenol or catechol fragments were characterized with more marked antioxidant properties than complexes **1**–**3**.

The antiproliferative activity of compounds **1**–**5** was studied in vitro against MCF-7, HTC-116, and A-549 cell lines by an MTT test. It was established that the presence trifluoromethyl substituents in ligands, as well as ethyl groups at tin atoms for complexes **1**–**3,** can cause a lowering of the IC_50_ parameter. Additionally, the values of IC_50_ are significantly affected by the presence of free antioxidant fragments and the coordination site for binding in the case of **4** and **5**. Thus, the synthesized compounds possess a dual anti/prooxidant activity in the dependence of the radical species generated in the assays. These compounds contain not only organometallic fragments but ligands with antioxidant groups, which can modulate the occupancy of tin atom coordination spheres and the bioactivity of such types of compounds.

## Figures and Tables

**Table 1:** The values of IC_50_ in the DPPH test (CH_2_Cl_2_, 298 K), the index of IC_50_, and TEAC (Trolox equivalents) in the ABTS^∙+^ assay and superoxide radical anion scavenging activity (NBT-test) in IC_50_ values of complexes **1**–**5** and ligands.

N	Compound	IC_50_ (DPPH), µM	IC_50_ (ABTS^∙+^), µM	ABTC_TEAC_	IC_50_ (O_2_^∙−^), µM
**1**	Ph_2_SnL^1^	>200	28.8 ± 0.9	0.62 ± 0.09	7.80 ± 0.23
**2**	Ph_2_SnL^2^	>200	25.0 ± 1.3	0.79 ± 0.11	5.16 ± 0.08
**3**	Et_2_SnL^2^	143.7 ± 10.3	35.4 ± 1.8	0.57 ± 0.04	24.02 ± 0.10
**4**	Ph_3_SnL^3^	44.7 ± 1.1	17.2 ± 0.5	1.25 ± 0.11	pr.a *
**5**	Ph_3_SnL^4^	16.1 ± 0.4	22.1 ± 0.7	0.93 ± 0.13	pr.a *
	L^1^H	42.1 ± 1.9	16.9 ± 0.8	1.02 ± 0.10	89.06 ± 3.10
	L^2^H	43.5 ± 1.5	14.2 ± 0.6	1.16 ± 0.12	74.07 ± 1.06
	L^3^H	30.0 ± 1.3	17.7 ± 1.2	1.07 ± 0.09	>100
	L^4^H	10.0 ± 0.5	8.8 ± 0.6	1.88 ± 0.10	68.69 ± 1.72
	Trolox	12.0 ± 0.5	16.0 ± 1.0	1.00 ± 0.03	62.7 ± 0.60

* pr.a—promotion action.

**Table 2:** The cytotoxic effects (IC_50_, µM) of complexes **1**–**5** against human cancer cell lines.

N	Compound	IC_50_, µM		
		A 549	HCT-116	MCF-7
**1**	Ph_2_SnL^1^	167.88 ± 9.32	54.6 ± 8.92	120.84 ± 5.44
**2**	Ph_2_SnL^2^	28.89 ± 1.15	11.56 ± 0.87	5.91 ± 0.03
**3**	Et_2_SnL^2^	72.06 ± 3.01	38.91 ± 1.74	75.09 ± 1.06
**4**	Ph_3_SnL^3^	0.42 ± 0.01	0.19 ± 0.02	0.23 ± 0.03
**5**	Ph_3_SnL^4^	0.32 ± 0.04	0.23 ± 0.01	0.31 ± 0.03
	^a^ L^3^H	30.63 ± 1.35	25.50 ± 0.99	43.19 ± 4.27
	^a^ L^4^H	3.53 ± 0.31	11.03 ± 0.17	7.43 ± 0.70
	^b^ cisplatin	9.02 ± 0.90	11.20 ± 1.90	15.2 ± 1.10
	Doxorubicin	0.13 ± 0.02	0.35 ± 0.05	0.62 ± 0.07

^a^ Data for ligands are from Ref. [42]; ^b^ data for cisplatin are from Ref. [43].

## Data Availability

The data presented in this study is available in this article.

## Supplementary Materials

The following supporting information can be downloaded at https://www.mdpi.com/article/10.3390/ijms24098319/s1.

## Sample Availability

Samples of the compounds are available from the authors.
