# Supplementary material for: The Synthesis and Biological Activity of Organotin Complexes with Thio-Schiff Bases Bearing Phenol Fragments
Source: Int J Mol Sci. 2023 May 5;24(9):8319. doi: 10.3390/ijms24098319 (PMC10179258; doi:10.3390/ijms24098319)
Supplement: Supplementary file 1 [file ijms-24-08319-s001.zip › ijms-2379392-supplementary.pdf]

Supplementary Information

Synthesis and Biological Activity of Organotin Complexes with thio-Schiff bases bearing Phenol Fragments

Ivan V. Smolyaninov, Andrey I. Poddel'sky, Daria A. Burmistrova, Yulia K. Voronina, Nadezhda P. Pomortseva, Maria A. Polovinkina, Nailya R. Almyasheva, Kristina S. Simeonova, Nadezhda T. Berberova and Igor L. Eremenko

Content:

Figure S1. ^{1}H NMR spectrum of **1** ($CDCl_3$, 400 MHz) 2

Figure S2. ^{13}C NMR spectrum of **1** ($CDCl_3$, 100 MHz) 2

Figure S3. ^{1}H NMR spectrum of **2** ($CDCl_3$, 400 MHz) 3

Figure S4. ^{13}C NMR spectrum of **2** ($CDCl_3$, 100 MHz) 3

Figure S5. ^{1}H NMR spectrum of **3** ($CDCl_3$, 400 MHz) 4

Figure S6. ^{13}C NMR spectrum of **3** ($CDCl_3$, 100 MHz) 4

Figure S7. ^{1}H NMR spectrum of **4** ($CDCl_3$, 400 MHz) 5

Figure S8. ^{13}C NMR spectrum of **4** ($CDCl_3$, 100 MHz) 5

Figure S9. ^{1}H NMR spectrum of **5** ($CDCl_3$, 400 MHz) 6

Figure S10. ^{13}C NMR spectrum of **5** ($CDCl_3$, 100 MHz) 6

Figure S11. ^{1}H NMR spectrum of **L^2H** ($CDCl_3$, 400 MHz) 7

Figure S12. ^{13}C NMR spectrum of **L^2H** ($CDCl_3$, 100 MHz) 7

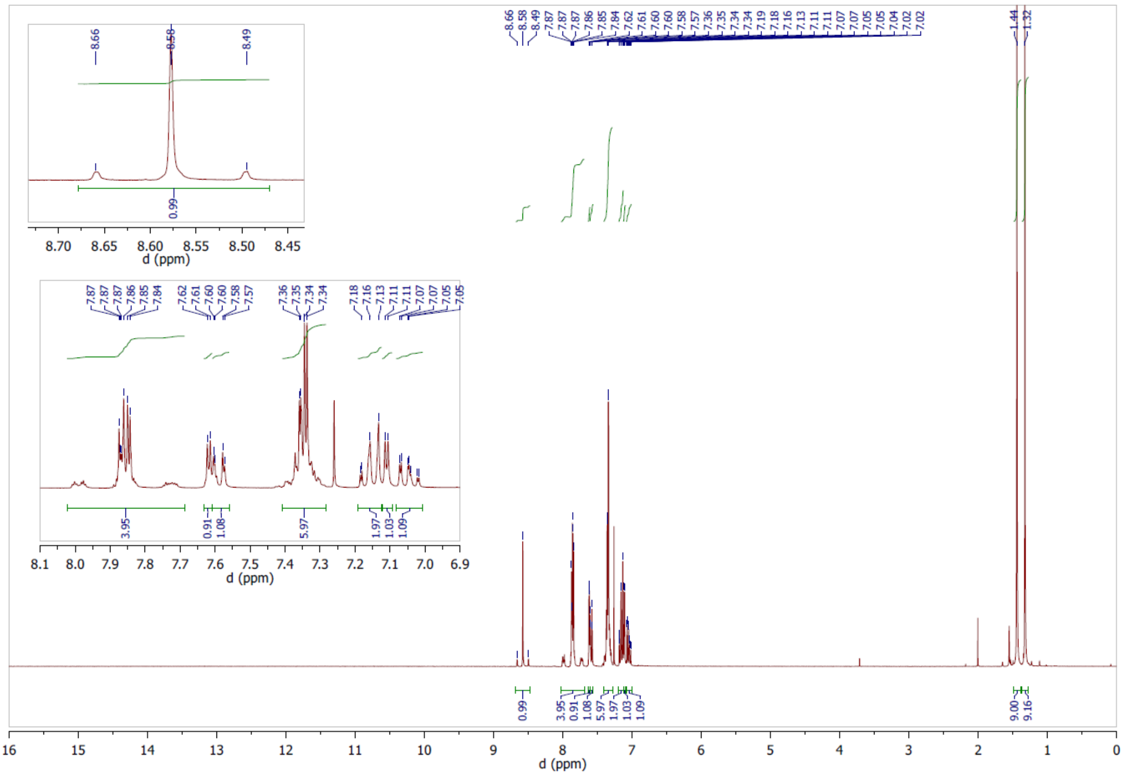

Figure S1. ^{1}H NMR spectrum of **1** ($CDCl_3$, 400 MHz)

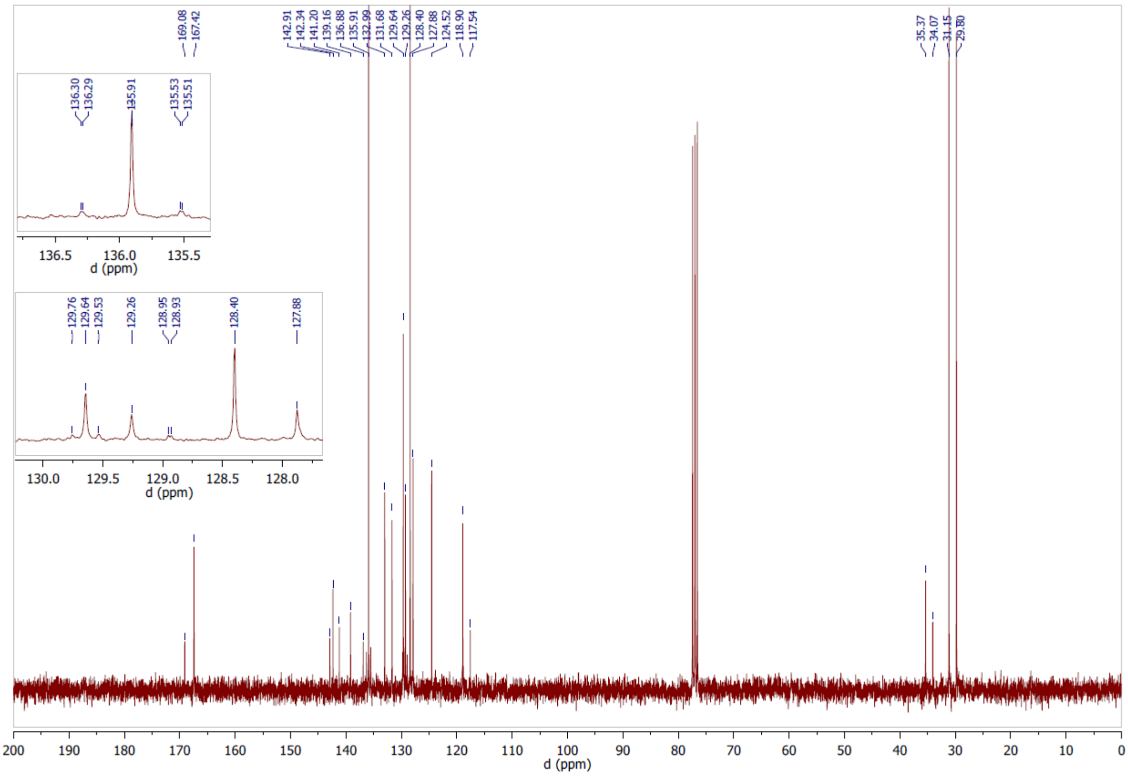

Figure S2. ^{13}C NMR spectrum of **1** ($CDCl_3$, 100 MHz)

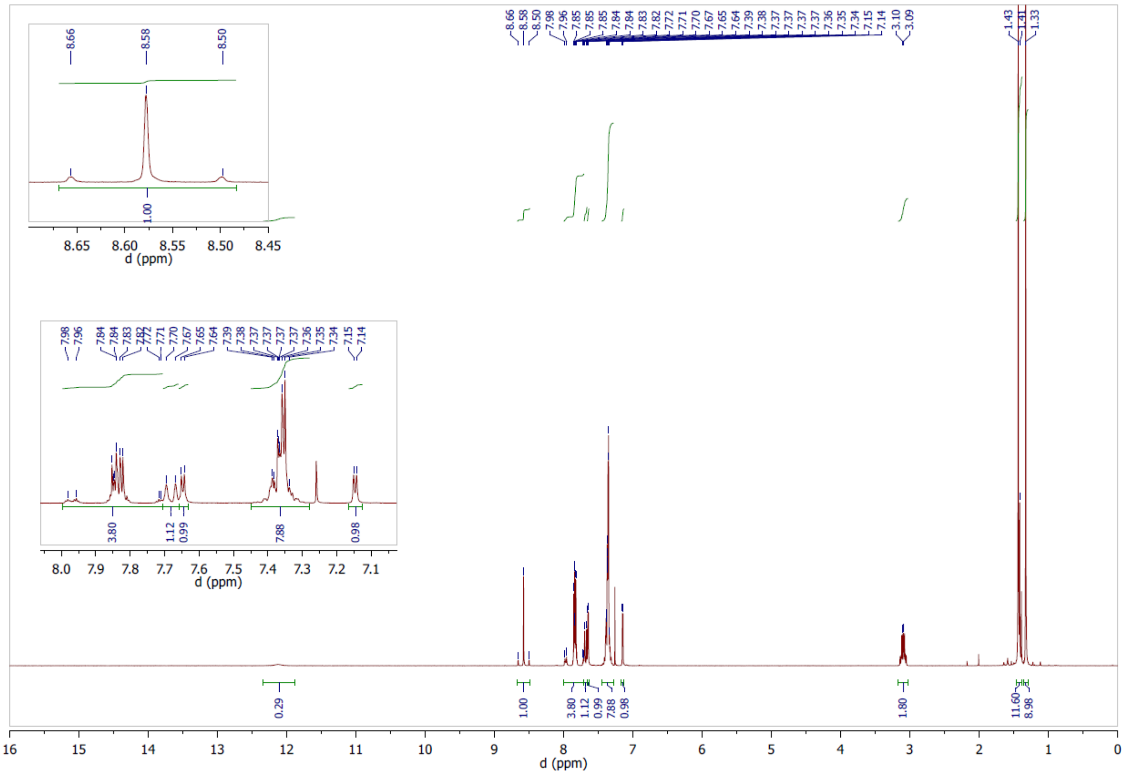

Figure S3. ^{1}H NMR spectrum of **2** ($CDCl_3$, 400 MHz). The signals at 1.41, 3.10, and 12.1 ppm are due to the admixture of $[Et_3NH]Cl$ (~ 0.3 mol per 1 mol of complex).

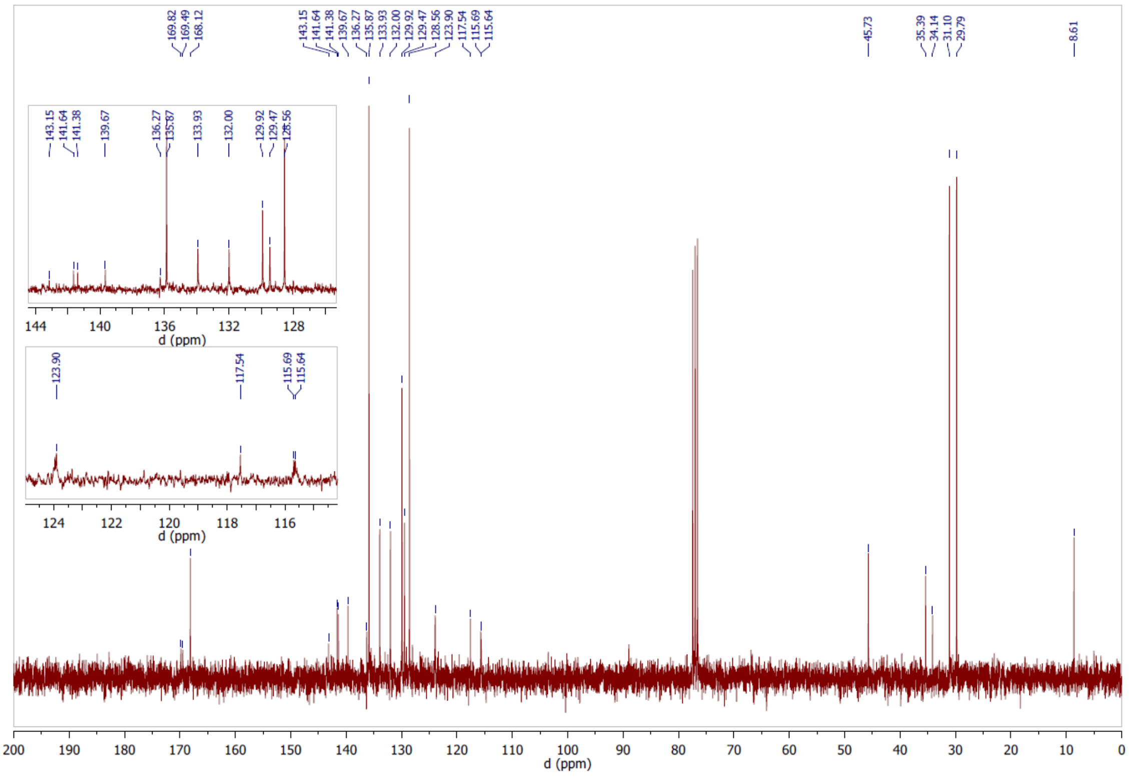

Figure S4. ^{13}C NMR spectrum of **2** ($CDCl_3$, 100 MHz). The signals at 8.61 and 45.73 ppm are due to the admixture of $[Et_3NH]Cl$ (~ 0.3 mol per 1 mol of complex).

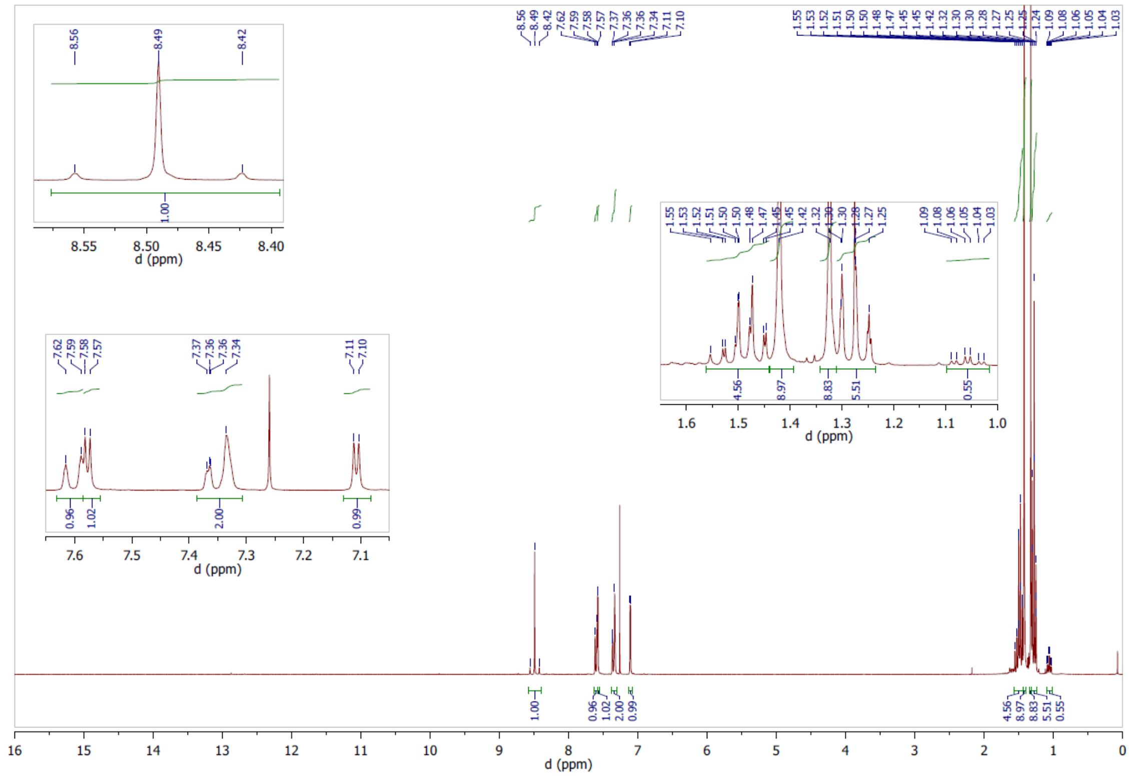

Figure S5. ^{1}H NMR spectrum of **3** ($CDCl_3$, 400 MHz)

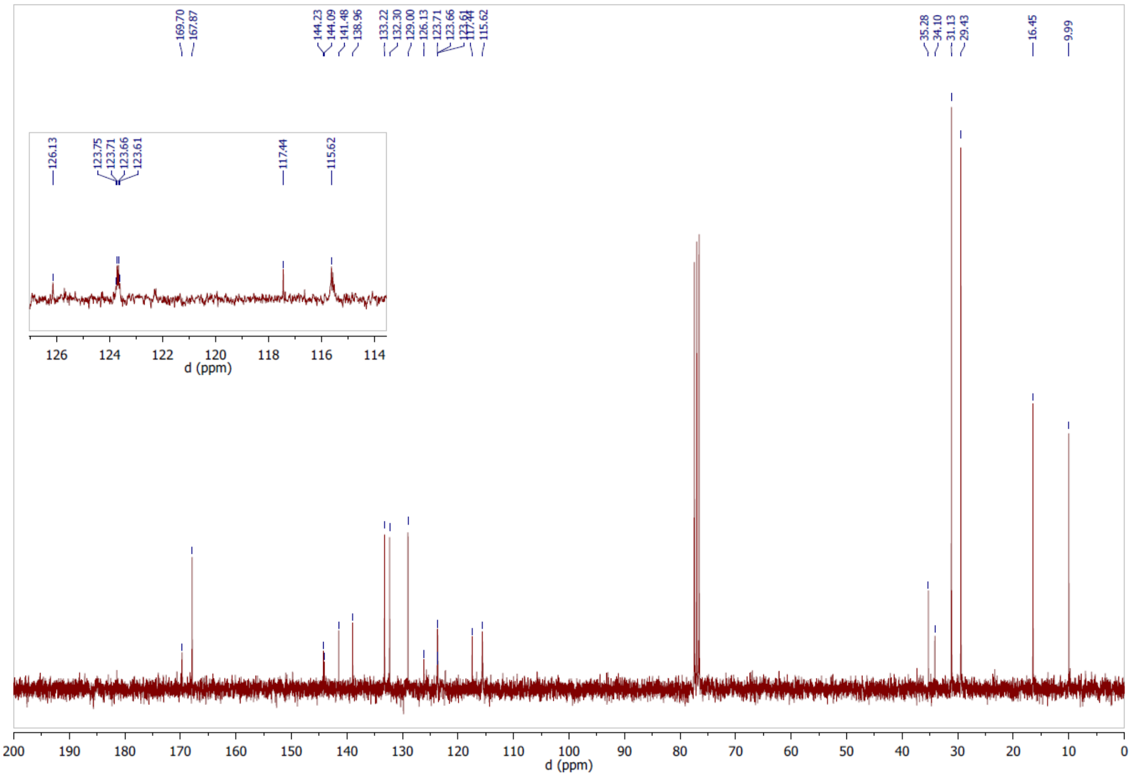

Figure S6. ^{13}C NMR spectrum of **3** ($CDCl_3$, 100 MHz)

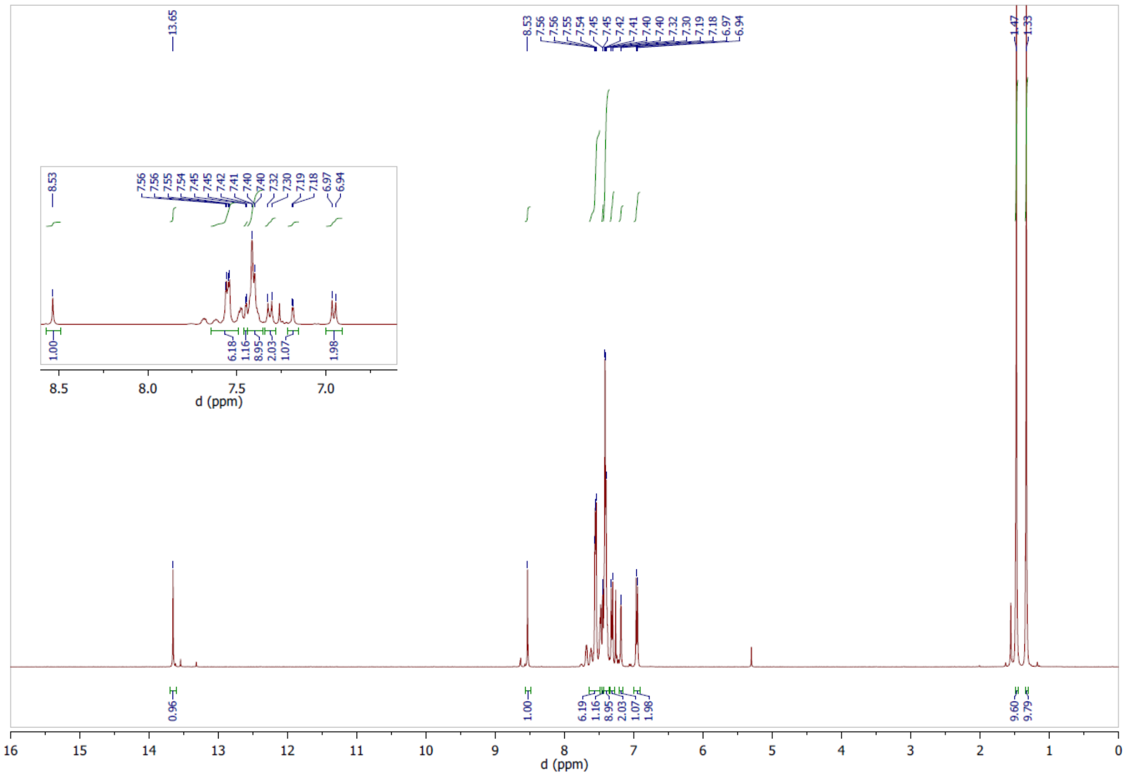

Figure S7. ^{1}H NMR spectrum of **4** ($CDCl_3$, 400 MHz)

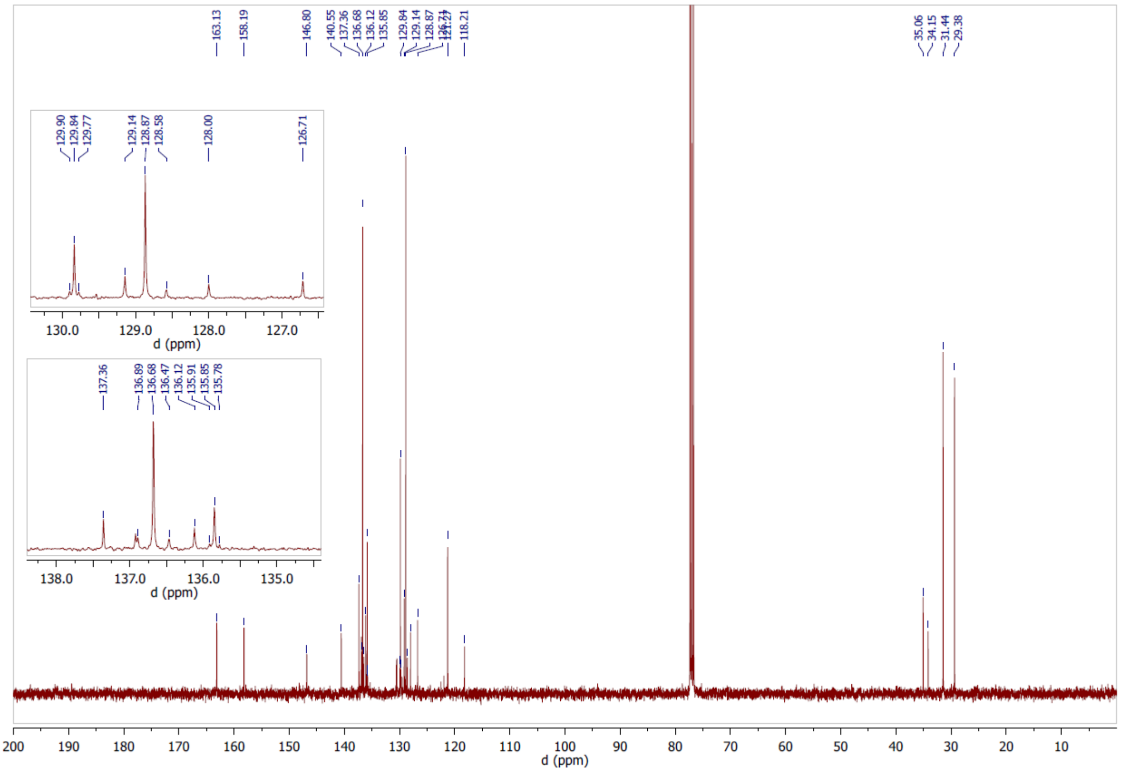

Figure S8. ^{13}C NMR spectrum of **4** ($CDCl_3$, 100 MHz)

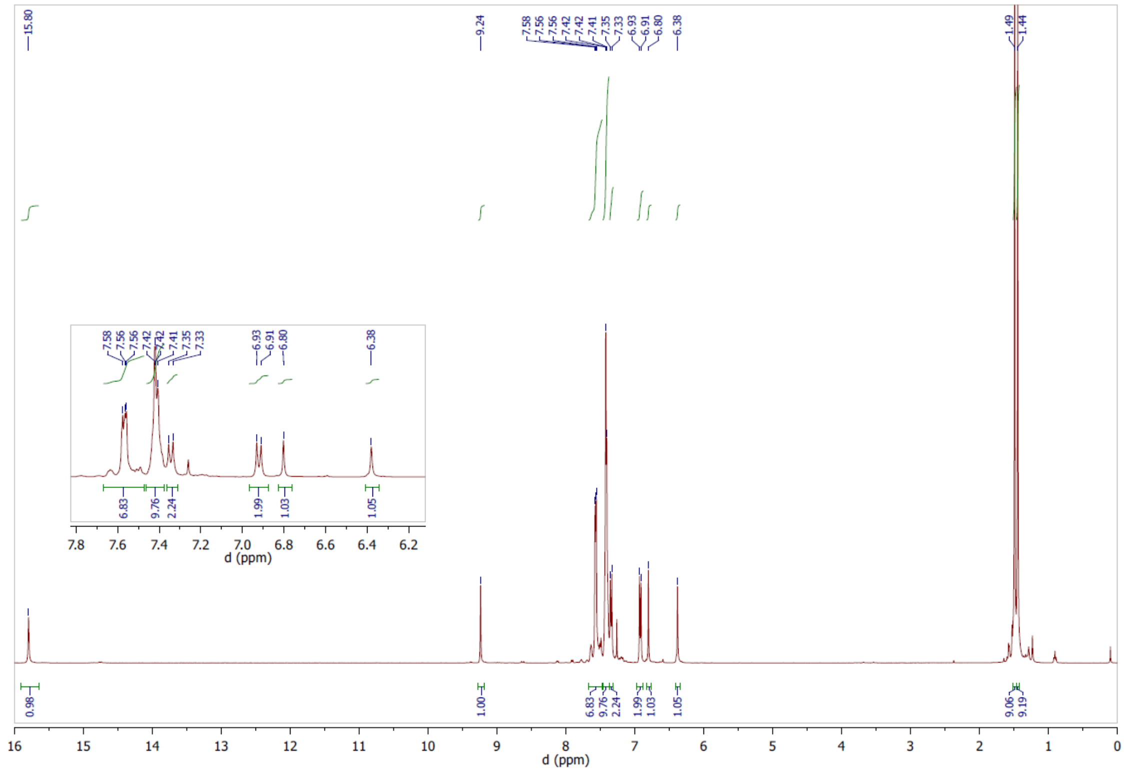

Figure S9. ^{1}H NMR spectrum of **5** ($CDCl_3$, 400 MHz)

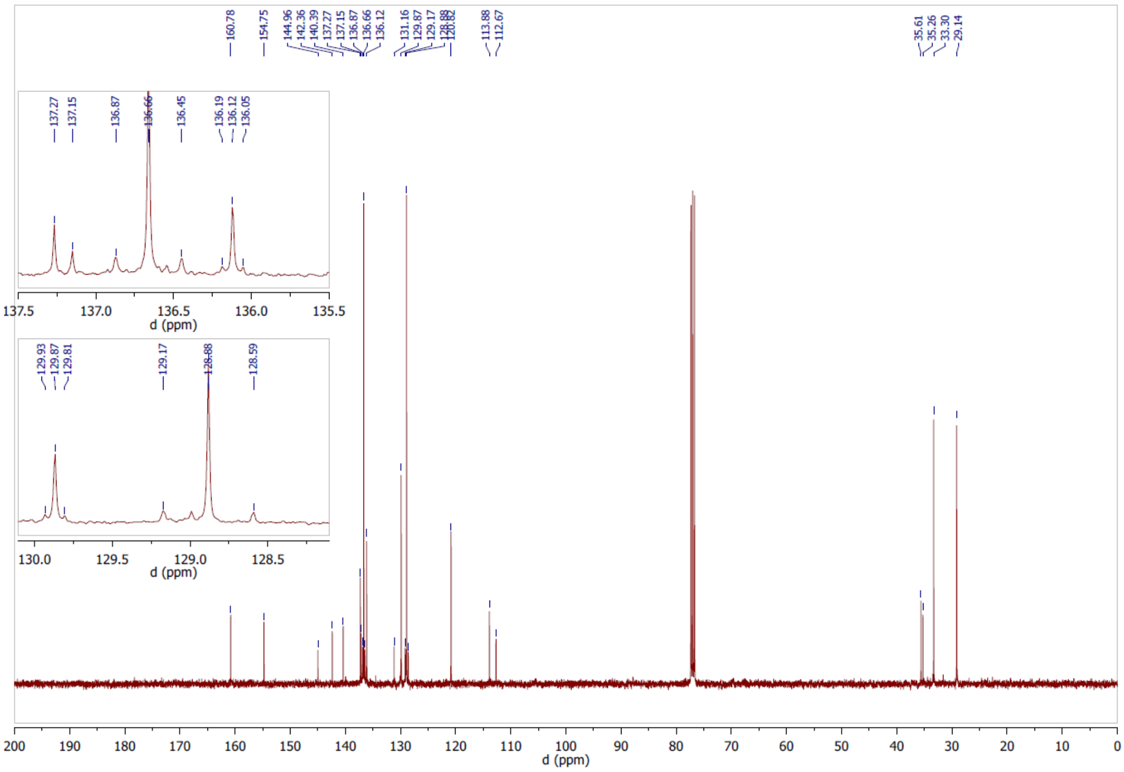

Figure S10. ^{13}C NMR spectrum of **5** ($CDCl_3$, 100 MHz)

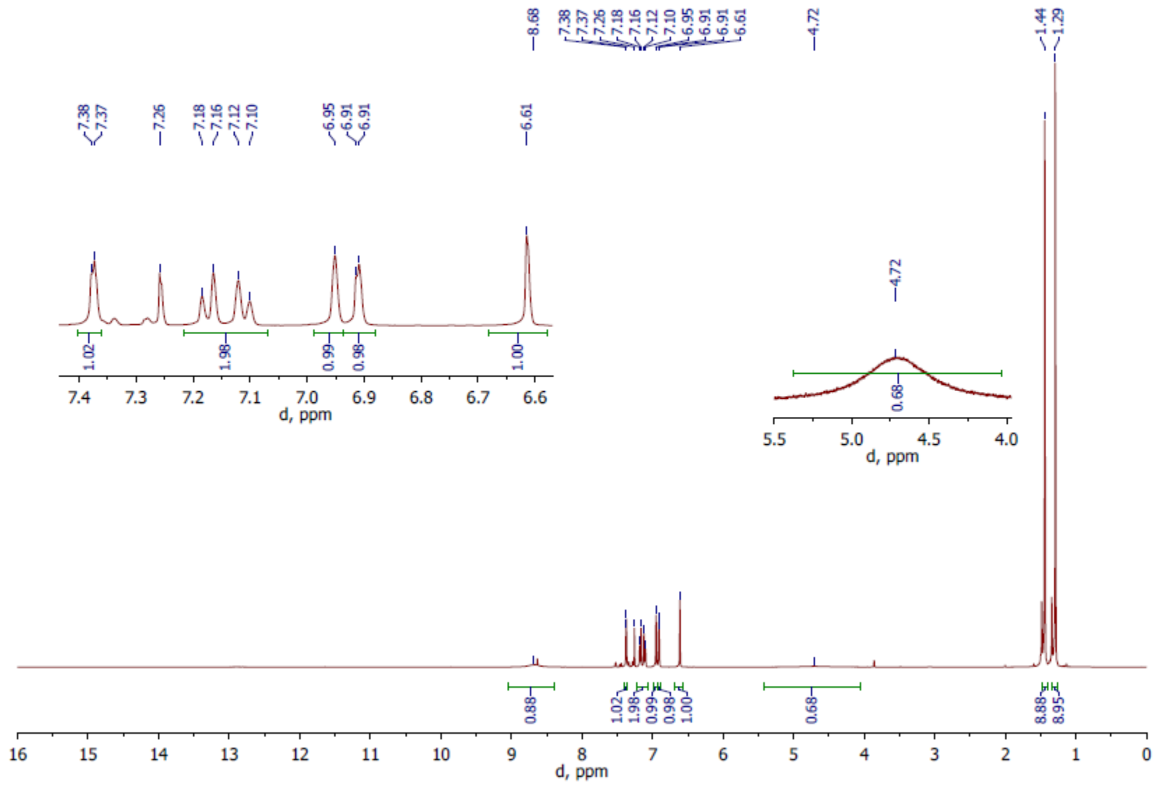

Figure S11. ^{1}H NMR spectrum of **L^{2}H** ($CDCl_3$, 400 MHz)

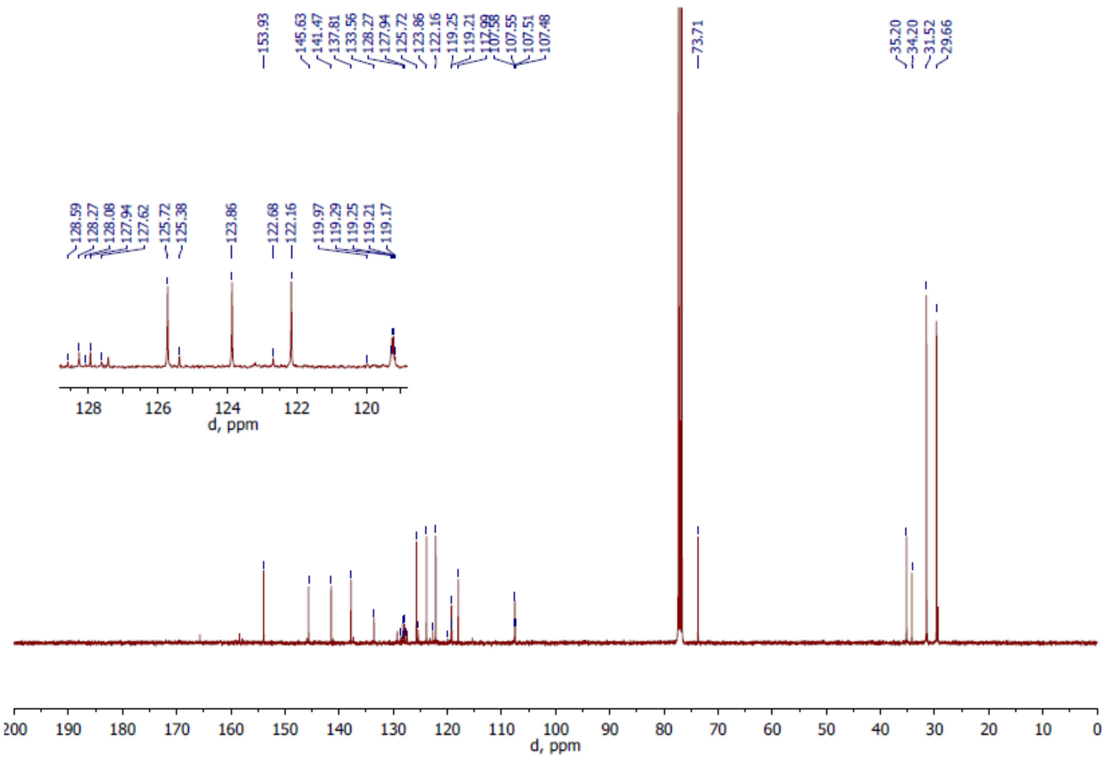

Figure S12. ^{13}C NMR spectrum of **L^{2}H** ($CDCl_3$, 100 MHz)
